# Hepatoprotective Effects of *Morchella esculenta* against Alcohol-Induced Acute Liver Injury in the C57BL/6 Mouse Related to Nrf-2 and NF-*κ*B Signaling

**DOI:** 10.1155/2019/6029876

**Published:** 2019-07-17

**Authors:** Bo Meng, Yuanzhu Zhang, Zhuqian Wang, Qinran Ding, Jia Song, Di Wang

**Affiliations:** ^1^State Key Laboratory of Food Nutrition and Safety, Tianjin University of Science & Technology, Tianjin 300457, China; ^2^Key Laboratory for Molecular Enzymology and Engineering of Ministry of Education, School of Life Sciences, Jilin University, Changchun 130012, China; ^3^Jilin Provincial Key Laboratory of Animal Embryo Engineering, Institute of Zoonosis, College of Animal Sciences, Jilin University, 130062 Changchun, Jilin Province, China

## Abstract

This study investigated the hepatoprotective effects of *Morchella esculenta* fruit body (ME) and the underlying mechanisms in mice with alcohol-induced acute liver injury. Systematic analysis revealed that ME contained 21 types of fatty acid, 17 types of amino acid, and 12 types of mineral. Subsequently, a mouse model of acute alcohol-induced liver injury was established by oral administration of alcohol for 14 days. Fourteen-day administration of ME prevented alcohol-induced increases in alanine aminotransferase and aspartate aminotransferase levels and reduced the activity of acetaldehyde dehydrogenase in blood serum and liver tissue. ME appears to regulate lipid metabolism by suppressing triglycerides, total cholesterol, and high-density lipoprotein in the liver. ME inhibited the production of inflammatory factors including chitinase-3-like protein 1 (YKL 40), interleukin-7 (IL-7), plasminogen activator inhibitor type 1 (PAI-1), and retinol-binding protein 4 (RBP4) in blood serum and/or liver tissue. ME treatment relieved the alcohol-induced imbalance in prooxidative and antioxidative signaling via nuclear factor-erythroid 2-related factor 2 (Nrf-2), as indicated by upregulation of superoxide dismutase-1, superoxide dismutase-2, catalase, heme oxygenase-1, and heme oxygenase-2 expression and downregulation of kelch-like ECH-associated protein 1 (Keap-1) in the liver. Moreover, ME reduced the levels of phosphorylated nuclear factor kappa-B kinase *α*/*β*, inhibitor of nuclear factor kappa-B *α* and nuclear factor kappa-B p65 (NF-*κ*B p65) in the liver. The hepatoprotective effects of ME against alcohol-induced acute liver injury were thus confirmed. The mechanism of action may be related to modulation of antioxidative and anti-inflammatory signaling pathways, partially via regulation of Nrf-2 and NF-*κ*B signaling.

## 1. Introduction

Alcoholic liver disease caused by alcohol abuse is a risk factor for chronic liver disease, leading to significant mortality worldwide [1]. Most patients are initially diagnosed with alcoholic fatty liver (AFL), which then develops further into alcoholic steatohepatitis (ASH). Chronic ASH eventually leads to liver fibrosis and cirrhosis and, in some cases, even acute hepatic failure (AHF) or death [2].

Much of the damage inflicted by alcohol abuse occurs in the liver. Approximately 90% of alcohol is oxidized in the liver, with only a small proportion being metabolized by alcohol dehydrogenase (ADH) and acetaldehyde dehydrogenase (ALDH) to form carbon dioxide and water for excretion [3]. Large amounts of alcohol can induce high activity of cytochrome P450 enzymes in the liver, leading to the overaccumulation of reactive oxygen species (ROS), resulting in lipid peroxidation [4]. Acetaldehyde, an intermediate of alcohol metabolism, is converted into superoxide and oxygen free radicals by xanthine oxidase, which destroys the structure and function of hepatocytes [5, 6]. During this process, oxidative stress-related liver damage occurs rapidly.

Nuclear factor-erythroid 2-related factor-2 (Nrf-2), one of the key intracellular antioxidant factors, regulates oxidative stress [7]. Nuclear factor-kappa B (NF-*κ*B), a transcription factor, is a crucial regulator of inflammation, immunity, and cell survival [8]. During ROS-mediated oxidative stress, activated NF-*κ*B is responsible for the transcription of various genes that mediate inflammatory cell recruitment, triggering a series of inflammatory reactions [9]. Promoting the activation of Nrf-2 signaling can regulate the redox balance in the body, inhibiting the activation of NF-*κ*B signaling and alleviating the oxidative stress-mediated inflammatory response.

Naltrexone, an opioid receptor antagonist commonly used for alcohol abuse treatment, has been shown to reduce hepatocellular damage in fibrotic animal models [10]; however, adverse effects, including anxiety, irritability, abdominal pain, nausea, and/or vomiting, have been reported [11]. Some edible mushrooms have a demonstrated ability to scavenge free radicals and mediate anti-inflammatory effects, making them candidates for the prevention and/or treatment of alcoholic liver disease [12]. *Morchella esculenta*, which contains various nutrients, has a variety of pharmacological activities including antioxidative, anti-inflammatory, and antitumor effects [13, 14]. A heteropolysaccharide isolated from the fruiting bodies of *M. esculenta* can effectively scavenge hydroxyl and superoxide radicals [15] and exert anti-inflammatory effects via blocking the activation of NF-*κ*B signaling [16]. Meanwhile, the total flavones of a fermentation broth resulting from coculture of *M. esculenta* and *Coprinus comatus* showed an anti-inflammatory effect on lipopolysaccharide-stimulated RAW264.7 macrophages via the mitogen-activated protein kinase (MAPK) signaling pathway [17]. Although the hepatoprotective effects of cultured *M. esculenta* mycelium against CCl_4_ and alcohol-induced chronic hepatotoxicity have been reported previously [18], protection engendered by its fruit body and the possible mechanism of action against alcohol-induced acute liver injury have not previously been investigated.

In this study, we systematically analyzed the components of the *M. esculenta* fruit body and verified its hepatoprotective effects in mice with alcohol-induced acute liver injury and then investigated the possible mechanisms related to the activation of Nrf-2 and NF-*κ*B signaling pathways.

## 2. Materials and Methods

### 2.1. *M. esculenta* Composition Analysis

The *M. esculenta* fruit bodies (ME) were collected from Dali, Yunnan, China, from April to June 2017. ME was dried at 60°C, crushed with a pulverizer, sieved through a 60-mesh sieve, and stored in a dryer for subsequent experiments.

#### 2.1.1. Main Component Analysis

The main components of ME including total sugar, reducing sugar, protein, total ash, crude fat, total flavonoids, total triterpenes, mannitol, and crude fiber were determined by the phenol sulfuric acid method [19], 3,5-dinitrosalicylic acid (DNS) reducing sugar assay [20], Kjeldahl method [21], combustion method [22], Soxhlet extraction [23], UV spectrophotometric detection, vanillin-glacial acetic acid and perchloric acid colorimetric method [24], high-performance liquid chromatography (HPLC) [25], and acid-base treatment, respectively.

#### 2.1.2. Fatty Acid Analysis

A 5% KOH-methanol solution was added to the ME powder, placed in a 60°C water bath for 30 min, and then mixed with 14% BF3-methanol solution at 60°C for 3 min. The samples were then mixed with hexane, and the levels of fatty acids were analyzed using a gas chromatography-mass spectrometer (QP2010, Shimadzu, Japan).

#### 2.1.3. Amino Acid Analysis

The ME was hydrolyzed using 6 mol/L HCl at 110°C ± 1°C for 22 h. After vacuum drying, the sample was dissolved in 1 mL of a pH 2.2 buffer. The amino acid content was quantified using an automatic amino acid analyzer (L-8900, Hitachi, Japan).

#### 2.1.4. Mineral Analysis

The ME was pretreated with hydrogen nitrate at a temperature of 110°C and an atmospheric pressure of 30 atm for 30 min. The levels of mercury (Hg), lead (Pb), selenium (Se), arsenic (As), cadmium (Cd), zinc (Zn), iron (Fe), manganese (Mn), chromium (Cr), calcium (Ca), copper (Cu), sodium (Na), and potassium (K) were detected by inductively coupled plasma optical emission spectrometry [26].

### 2.2. Animal Models and Treatment

Sixty healthy male C57BL/6 mice (8 weeks old, 18–22 g) (SCXK (LIAO) 2015-0001) were purchased from Liaoning Changsheng Biotechnology Co. Ltd. (Liaoning, China). Animal care and experimental protocols were approved by the Institutional Animal Ethics Committee of Jilin University (20170106). The mice were acclimatized for 7 days before the experiments under a controlled environment at a temperature of 23°C ± 1°C with 50% ± 10% humidity and a 12 h light-dark cycle and *ad libitum* access to water and food.

The methodology for establishing the mice model with alcohol-induced acute liver injury was modified according to previous studies [27, 28]. The mice were randomly divided into six groups (*n* = 10/group). Ten control mice were administered 0.9% physiological saline intragastrically twice per day. The other 50 mice were administered alcohol intragastrically at a dose of 13 g/kg (Beijing Shunxin Agricultural Co. Ltd., China) at 09:00 once per day for 14 days. On the same days, at 16:00, the alcohol only treated mice (the model group) were administered physiological saline intragastrically at a dose of 0.1 mL/10 g. The positive control mice were administered silybin (Sil), a clinical used liver protectant, which can reduce oxidative stress in patients with alcoholic liver disease and prevent the lipid peroxidation [29], intragastrically at a dose of 60 mg/kg (Tianjin Tasly Sants Pharmaceutical Co. Ltd., China).The ME-treated mice were administered ME orally at a dose of 200 mg/kg, 400 mg/kg, or 800 mg/kg, respectively. The ME was pulverized using an ultrafine grinder (XDW-6A, Jinan Tatsu Micro Machinery Co. Ltd., Jinan, China) and suspended in physiological saline. Before administration, the mixture was shaken. During the experimental period, the mice were weighed daily. Following the last administration, all mice were fasted overnight, and blood samples were collected from the caudal vein of each mouse. All mice were then euthanized by CO_2_ inhalation, and their livers, kidneys, and spleens were removed for further analysis.

### 2.3. Biochemical Parameter Assays

The collected liver and spleen samples from experimental mice were homogenized in physiological saline on ice using a hand-held homogenizer (LEOPARD Scientific Instruments Co. Ltd., Beijing, China), and then the supernatant was taken by centrifugation at 3500 rpm for 10 minutes. The levels of gamma-glutamyl transferase (GGT) (MM-43976M1), 8-hydroxydeoxyguanosine (8-OHdG) (MM-0221M1) (Jiangsu FEIYA Biotechnology Co. Ltd., Jiangsu, China), aspartate aminotransferase (AST) (CK-E90386M), alanine aminotransferase (ALT) (CK-E90314M), aldehyde dehydrogenase (ALDH) (CK-E92649M), chitinase-3-like 1 protein 1 (YKL 40) (CK-E95772M), interleukin-7 (IL-7) (CK-E20125M), plasminogen activator inhibitor type 1 (PAI-1) (CK-E93562M), and retinol-binding protein 4 (RBP4) (CK-E20170M) in the serum and liver; the levels of reactive oxygen species (ROS) (CK-E91516M), malondialdehyde (MDA) (CK-E20347M), nitric oxide (NO) (CK-E20293M), superoxide dismutase (SOD) (CK-E20348M), glutathione peroxidase (GSH-Px) (CK-E92669M), and catalase (CAT) (CK-E92636M) in the serum, liver, and spleen; and the levels of high-density lipoprotein (HDL) (CK-E91912M), total cholesterol (TC) (CK-E91839M), and triglyceride (TG) (CK-E91733) (Shanghai Yuanye Biological Technology Co. Ltd., Shanghai, China) in the liver were detected using the enzyme-linked immunosorbent assay (ELISA) kits according to the manufacturers' instructions.

### 2.4. Histopathological Analysis

Liver and kidney tissue samples were fixed in 10% (*v*/*v*) formalin buffer and embedded in paraffin. The paraffin sections were sliced into thicknesses of 4 *μ*m to 6 *μ*m, and each section was stained with hematoxylin and eosin (H&E) for histological evaluation. The pathological sections were observed under a light microscope (Olympus, Japan) and photographed.

### 2.5. Western Blot Analysis

Samples of liver tissue collected from each mouse were homogenized in radioimmunoprecipitation assay (RIPA) buffer (Sigma-Aldrich, USA) containing 2% phenylmethanesulfonyl fluoride (PMSF) (Sigma-Aldrich, USA) and 1% protease inhibitor cocktail (Sigma-Aldrich, USA). The protein concentration of the homogenate was determined using a bicinchoninic acid (BCA) protein assay kit (Merck Millipore, Germany). 40 *μ*g of protein was electrophoretically separated in a 12% polyacrylamide gel and transferred to 0.45 *μ*m polyvinylidene fluoride (PVDF) membranes (Millipore, Germany). The membranes were blocked with 5% bovine serum albumin (BSA) blocking solution (Sigma, USA) at 4°C for 4 h. The blocked membranes were incubated with primary antibodies at a dilution of 1 : 1000 against phosphor- (P-) inhibitor of nuclear factor kappa-B kinase *α*/*β* (IKK*α*/*β*, ab195907), total- (T-) IKK*α*/*β* (ab178870), P-inhibitor of nuclear factor kappa-B kinase *α* (I*κ*B*α*) (ab12135), T-I*κ*B*α* (ab32518), P-NF-*κ*B p65 (ab86299), T-NF-*κ*B p65 (ab7970), manganese superoxide dismutase 2 (SOD-2) (ab13533), heme oxygenase-1 (HO-1) (ab137749), heme oxygenase-2 (HO-2) (ab90492), Nrf-2 (ab137550), kelch-like ECH-associated protein 1 (Keap-1) (ab66620), CAT (ab16731) (Abcam, Cambridge, USA), SOD-1 (bs-10216R) (Bioss, China), and glyceraldehyde-3-phosphate dehydrogenase (GAPDH) (ABS16) (Millipore, Germany) at 4°C overnight. After incubation, the membranes were washed five times with 1x TBS containing 0.1% Tween 20 for 5 minutes each time. The membranes were then incubated with anti-mouse (IH-0031) and anti-rabbit (IH-0011) (Dingguo, Beijing, China) horseradish peroxidase-conjugated secondary antibodies for 4 h at 4°C. The target proteins were visualized using Immobilon Western Chemiluminescent HRP Substrate (WBKLS0500, Millipore, Germany) and a gel imaging system (UVP, USA). Images were quantified with ImageJ analysis software version 1.46 (National Institutes of Health, Bethesda, MD).

### 2.6. Statistical Analysis

All data were expressed as the mean ± standard error of the mean (S.E.M.). Differences were determined by one-way analysis of variance followed by post hoc multiple comparisons (Holm-Sidak test) using SPSS 23.0 software (IBM Corporation, Armonk, USA). Statistical significance was declared for *p* values under 0.05.

## 3. Results

### 3.1. Composition of ME

Table 1 shows the details of the components of the ME. It was found to contain 8.1% total sugar, 2.8% reducing sugar, 34.8% protein, 8.0% total ash, 9.0% crude fat, 1.3% total triterpenes, 1.8% mannitol, 0.4‰ crude fiber, and 0.5‱ total flavonoids. Twenty-one fatty acids and 17 amino acids were detected, with the content of aspartic acid, glutamic acid, and arginine being higher than that of other amino acids. Thirteen minerals were detected, with the levels of Hg, Pb, As, and Cd falling below the detection limit.

### 3.2. The Hepatoprotective Effects and Regulation of Lipid Metabolism by ME in Mice with Alcohol-Induced Acute Liver Injury

**Figure 1:**
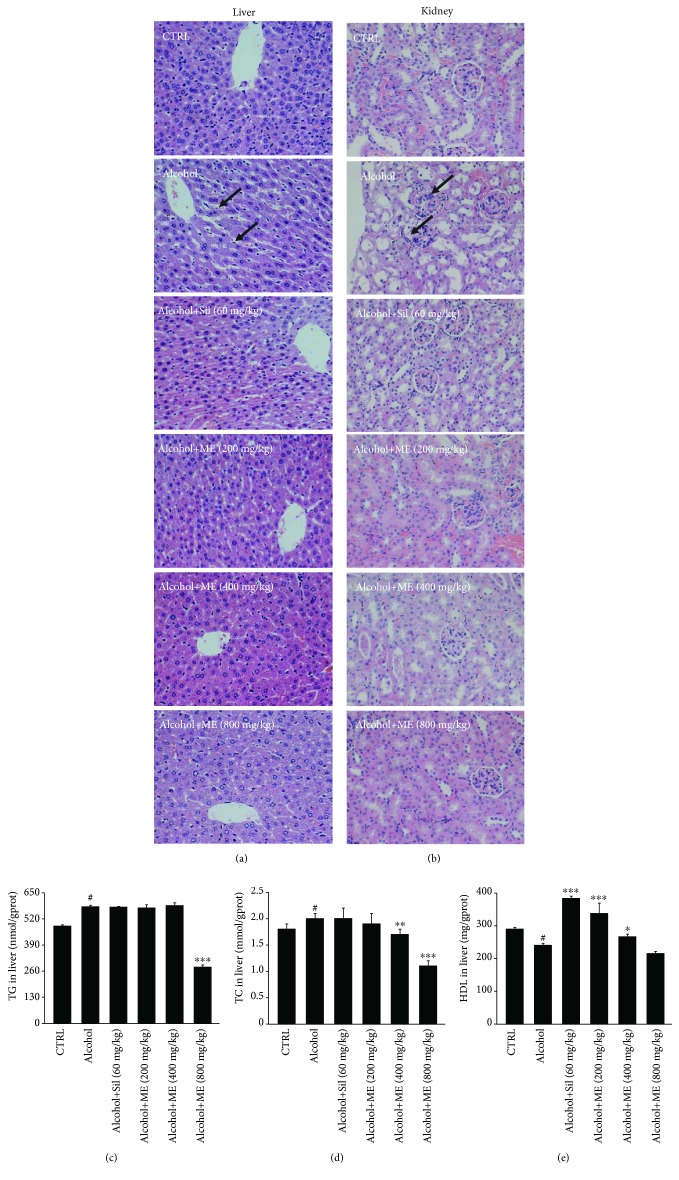
The hepatoprotective effects of ME in mice with acute alcohol injury. Mice were treated with 13 g/kg of alcohol and/or cotreated with 60 mg/kg of Sil or 200, 400, or 800 mg/kg of ME for 14 days. Histopathological analysis of (a) liver and (b) kidney analyzed with H&E staining (40x). In livers, arrows showed the inflammation infiltration and the fat droplet. In kidneys, arrows showed the inflammation infiltration and the swelling glomerulus. ME and Sil reduced the levels of (c) TG and (d) TC and (e) increased the levels of HDL in the livers of mice with alcohol-induced acute liver injury. Data are expressed as mean ± S.E.M. (*n* = 10). ^#^ *p* < 0.05 vs. control mice, ^∗^ *p* < 0.05, ^∗∗^ *p* < 0.01, and ^∗∗∗^ *p* < 0.001 vs. alcohol only treated mice. ME: *M. esculenta*, Sil: silybin.

The reduction in body weight seen in the mice treated with alcohol was strongly prevented by ME but not by Sil (*p* < 0.05, Table 1S). ME-only treatment had no effects on the body weight of healthy mice (Fig.  1S A).

The serum and liver tissue levels of AST, ALT, and GGT, which are important indexes of injury, were increased by alcohol treatment, and ME treatment prevented these increases (*p* < 0.05, Table 2). Sil reduced the levels of GGT in the serum and liver (*p* < 0.05, Table 2). ME alone had no effect on the serum levels of AST (Fig.  1S B) and ALT (Fig.  1S C).

In alcohol metabolism, ALDH helps to convert harmful acetaldehyde into harmless acetic acid [30]. Both ME and Sil prevented the decline in levels of ALDH in the serum and liver of acute alcohol-exposed mice (*p* < 0.05, Table 2). ME alone had no effect on the serum levels of ALDH (Fig.  1S D).

ME and Sil strongly alleviated the loose arrangement of hepatocytes and prevented inflammatory cell infiltration and the formation of fat droplets in liver tissues of acute alcohol-exposed mice (Figure 1(a)). In the kidney, the inflammation infiltration and swelling of the glomerulus caused by alcohol were significantly relieved by ME and Sil treatment (Figure 1(b)).

Lipid metabolism, especially the levels of TG, TC, and HDL, can serve as a metric indicating the extent of liver injury [31]. Alcohol caused extremely high levels of TG and TC and low levels of HDL in the livers of the mice (*p* < 0.05, Figures 1(c)–1(e)). Compared with alcohol only treated mice, ME reduced the levels of TG and TC by 51.9% (*p* < 0.001, Figure 1(c)) and >15% (*p* < 0.01, Figure 1(d)), respectively, and enhanced the levels of HDL by >11.1% (*p* < 0.05, Figure 1(e)). Sil only enhanced the hepatic levels of HDL (*p* < 0.001, Figure 1(e)) but not TG or TC (Figures 1(c) and 1(d)).

### 3.3. The Anti-inflammatory Effects of ME in Mice with Alcohol-Induced Acute Liver Injury

In comparison with the alcohol only treated mice, ME prevented the increase in the serum and liver tissue levels of IL-7, YKL 40, and PAI-1 (*p* < 0.05, Table 3) and prevented the reduction in the levels of RBP4 in the liver (*p* < 0.01, Table 3), but had no effect on the levels of RBP4 in the serum (Table 3). Sil increased the levels of RBP4 and reduced the levels of IL-7, YKL 40, and PAI-1 in the liver (*p* < 0.05, Table 3), but only reduced the levels of YKL 40 and PAI-1 in the serum of mice with alcohol-induced acute liver injury (*p* < 0.05, Table 3).

### 3.4. Antioxidative Effects Are Involved in ME-Mediated Hepatoprotection

Oxidative stress is central to the development and progression of liver disease resulting from alcohol consumption. Excessive ROS can oxidize carbohydrates, proteins, and DNA molecules, resulting in damage to hepatocytes [32]. ME prevented an elevation in the levels of ROS, MDA, NO, and 8-OHdG and prevented a reduction in the SOD, GSH-Px, and CAT levels in the serum (*p* < 0.05, Table 4), livers (*p* < 0.05, Table 4), and spleens (*p* < 0.05, Table 2S) of mice with alcohol-induced acute liver injury. However, Sil failed to influence the levels of ROS, MDA, and NO in the serum and livers of alcohol-exposed mice (Table 4). In the spleen, Sil suppressed the levels of ROS and MDA and enhanced the levels of SOD and GSH-Px (*p* < 0.05, Table 2S).

### 3.5. ME Regulates the Activation of Nrf-2 and NF-*κ*B Signaling in the Livers of Alcohol-Treated Mice

**Figure 2:**
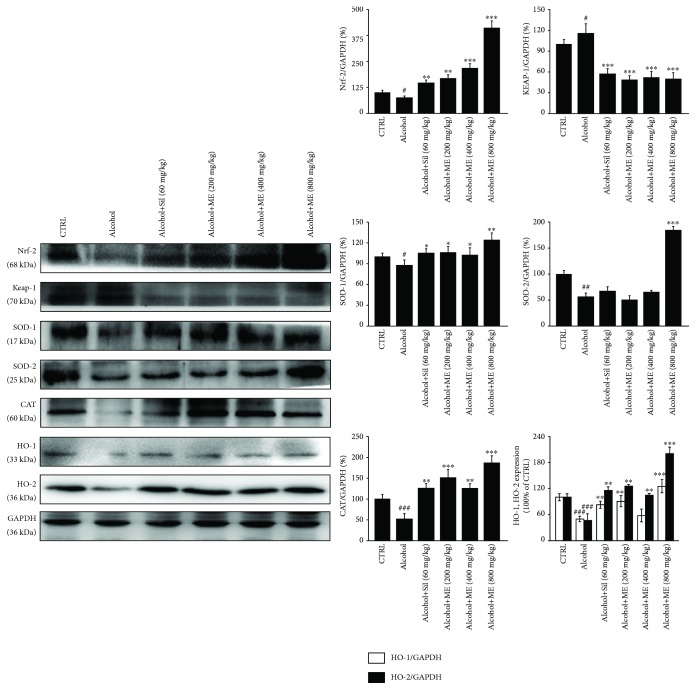
Nrf-2 signaling is involved in ME-mediated hepatoprotection against alcohol-induced acute liver injury. Fourteen-day ME and Sil administration reduced the expression levels of Keap-1 and enhanced the expression levels of Nrf-2 and its downstream proteins including CAT, HO-1, HO-2, SOD-1, and SOD-2 in the livers of alcohol-damaged mice. Protein expression levels were normalized to that of GAPDH. Data are expressed as mean ± S.E.M. (*n* = 6). ^#^ *p* < 0.05, ^##^ *p* < 0.01, and ^###^ *p* < 0.001 vs. control mice, ^∗^ *p* < 0.05, ^∗∗^ *p* < 0.01, and ^∗∗∗^ *p* < 0.001 vs. alcohol only treated mice. ME: *M. esculenta*, Sil: silybin.

High expression levels of Keap-1 and low expression levels of Nrf-2 and the downstream antioxidative stress factors were found in the livers of alcohol only treated mice (*p* < 0.05, Figure 2). Comparatively, ME administration strongly prevented these reactions to alcohol treatment, as indicated by the reduced expression levels of Keap-1 and increased levels of Nrf-2, SOD-1, SOD-2, CAT, HO-1, and HO-2 (*p* < 0.05, Figure 2). Sil exerted similar effects on the expression levels of Nrf-2 and its downstream proteins (*p* < 0.05, Figure 2) except for SOD-2 (Figure 2).

**Figure 3:**
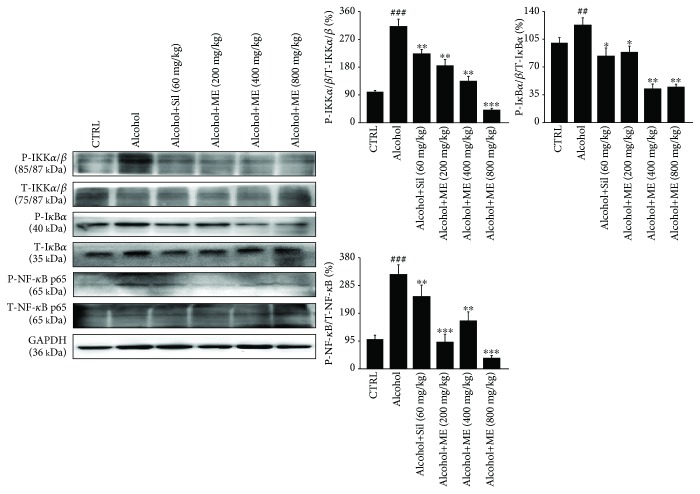
NF-*κ*B signaling is involved in ME-mediated hepatoprotection against alcohol-induced acute liver injury. Fourteen-day ME and Sil administration reduced the expression levels of P-IKK*α*/*β*, P-I*κ*B*α*, and P-NF-*κ*B p65 in the livers of alcohol-damaged mice. Phosphorylated protein expression levels were normalized to corresponding total protein expression levels. Data are expressed as mean ± S.E.M. (*n* = 6). ^##^ *p* < 0.01 and ^###^ *p* < 0.001 vs. control mice, ^∗^ *p* < 0.05, ^∗∗^ *p* < 0.01, and ^∗∗∗^ *p* < 0.001 vs. alcohol only treated mice. ME: *M. esculenta*, Sil: silybin.

NF-*κ*B, a key regulator involved in inflammation, controls the expression of proinflammatory genes and regulates the production of inflammatory factors, processes which are closely related to the pathogenesis of alcoholic liver disease [33]. Compared with the alcohol-treated mice, Sil and ME significantly reduced the phosphorylated levels of downstream factors IKK*α*/*β*, I*κ*B*α*, and NF-*κ*B p65 in liver tissues (*p* < 0.05, Figure 3).

## 4. Discussion

*M. esculenta* fruit bodies were found to contain 21 fatty acids, 17 amino acids, and 13 minerals, indicating the nutritive value of this fungus. The trace levels of heavy metals found suggest that it is safe for consumption. As previously reported, ME polysaccharides and proteins can increase antioxidant enzyme activity and improve immune system function [14, 34]. *M. esculenta* polysaccharide protects NR8383 cells from PM2.5-induced inflammation by inhibiting NF-*κ*B activation [16], and total flavonoids of *M. esculenta* have anti-inflammatory effects through lipopolysaccharide-stimulated RAW264.7 macrophages [17]. These nutritional components provide a foundation for understanding the hepatoprotective effects of ME in the treatment of alcohol-induced acute liver injury. The multieffective components may target many key molecules in the signaling pathways of inflammation and oxidative stress. This “systemic targeting” might eliminate inflammation and oxidative stress in a natural way, meaning less adverse side effects would be expected. Additionally, it helps to explain the non-dose-dependent activities of ME observed in most of our experiments, which is in fact a common feature of pharmaceutically active natural products [35].

AST and ALT are frequently elevated after excessive alcohol intake. When liver cells are damaged, cell membrane permeability increases and AST and ALT are released into the blood, increasing serum transaminase content, which is the essential enzyme in metabolic processes [36]. A high level of GGT can be detected in patients with liver disease and has been used as a sensitive indicator of liver injury [37]. Excessive alcohol consumption can result in overaccumulation of acetaldehyde in the liver, which promotes the formation of protein adducts through reactions with various macromolecules in the body, leading to functional impairment of key proteins [38]. ALDH, a key enzyme in the metabolism of alcohol, can catalyze acetaldehyde into acetic acid, allowing its excretion in the form of CO_2_ and H_2_O [39]. ME strongly prevented the pathologic changes in the levels of AST, ALT, GGT, and ALDH in alcohol-injured mice. However, it failed to influence the ratio of AST/ALT [40], providing a basis for further experiments.

Inflammation in the liver due to excessive alcohol consumption ultimately leads to fibrosis and impaired liver function [41]. RBP4, which possesses a nuclear transcription factor activator protein sequence [42], is mainly synthesized in the liver, and its expression can be reduced in acute liver disease, chronic hepatitis, and cirrhosis [43]. Increased expression of YKL-40 has been reported in fibrotic regions of the liver [44], and inhibitors of PAI-1 can alleviate this fibrosis [45]. The pathological alterations to the expression of these cytokines in mice chronically exposed to alcohol were prevented following 14-day ME administration, suggesting its hepatoprotective and anti-inflammatory properties. Furthermore, NF-*κ*B, serving as a key inflammatory response mediator, regulates multiple aspects of innate and adaptive immune function [46]. Typically, NF-*κ*B binds to the I*κ*B protein to form a complex that blocks the nuclear localization signal of NF-*κ*B. However, when the cells are stimulated by alcohol, the I*κ*B protein is phosphorylated by activated IKK, leading to the nuclear localization signal of NF-*κ*B being exposed, allowing transportation to the nucleus [47]. Nuclear NF-*κ*B mediates transcription of the IL-7 receptor *α*-subunit (CD127) [48] and YKL-40 [49] and regulates the expression of PAI-1 and RBP4 [42, 50].

Oxidative stress is one of the most important processes in the pathogenesis of alcoholic liver disease [51]. In this study, ME exerted a strong antioxidant effect through increasing the expression levels of antioxidative enzymes and reducing the levels of ROS, MDA, and 8-OHdG in alcohol-exposed mice. ME may regulate aspects of the immune system through modulation of oxidative stress-related factors in the spleen, an important immune organ. ROS, a strong oxidant, can damage cellular macromolecules such as DNA, lipids, and proteins and can also promote lipid peroxidation [52]. MDA, the end product of lipid peroxidation, can reflect the degree of lipid peroxidation, thus reflecting the extent of the damage of liver cells [53]. 8-OHdG is a widely accepted biomarker for reflecting the oxidative DNA damages [54]. Alcohol significantly destroys the antioxidant defense enzyme activity in the body. Under normal circumstances, SOD can convert superoxide into H_2_O_2_, while CAT can convert H_2_O_2_ into H_2_O [55]. When redox is imbalanced, the activity levels of the antioxidant enzymes are impaired. The transcription factor Nrf-2 is a major player in the control and expression of a panel of defense genes encoding antioxidant enzymes [56]. Under normal conditions, Nrf-2 binds to Keap-1 in the cytoplasm in an inactive form. When exposed to oxidative stress caused by alcohol, Nrf-2 dissociates from Keap-1 and transfers to the nucleus, where it interacts with antioxidant response elements (AREs). Subsequently, Nrf-2 mediates the expression of the downstream genes HO-1 and SOD-1, suggesting that the cytoprotective effect of Nrf-2 may be attributable to the induction of these enzymes [57]. As previously reported, oxidative stress can initiate or amplify inflammation by upregulating the genes involved in the inflammatory response through activation of NF-*κ*B signaling [58], and Nrf-2 can directly inhibit the expression of NF-*κ*B [59]. Our present data suggest that the protective effects of ME on hepatocytes may be related to its regulation of Nrf-2 signaling.

This study had some limitations. Although we found that ME can regulate the activation of Nrf-2 and NF-*κ*B signaling, the specific siRNA and/or related inhibitors were not used in the experimental model. The relationship between Nrf-2 and NF-*κ*B signaling remains to be elucidated. As previously reported, chronic severe alcohol consumption can increase intestinal permeability, promoting the activation of Kupffer cells, future triggering inflammation in the liver [60], which were failed to investigate in the present study. Furthermore, although ME and Sil displayed similar liver protection against acute alcohol damage in mice, it is hard to conclude which one shows better effects based on our present data. All of these issues, especially the mechanisms of ME-mediated hepatoprotection against alcohol-induced damage, will be further investigated by our group.

We have successfully confirmed the hepatoprotective effects of ME in alcohol-induced acute liver injury and found that this effect may be partially related to its ability to favorably modulate antioxidative anti-inflammatory signaling pathways through regulation of Nrf-2 and NF-*κ*B.

## Figures and Tables

**Table 1:** Composition of *Morchella esculenta* (ME).

	Compounds	Contents	Compounds	Contents	Compounds	Contents
Main components	Total sugar (%)	8.10	Reducing sugar (g/100 g)	2.80	Protein (g/100 g)	34.80
	Total ash (%)	7.96	Crude fat (g/100 g)	9.02	Total flavonoids (mg/kg)	53.64
	Total triterpenes (g/100 g)	1.28	Mannitol (g/100 g)	1.82	Crude fiber (%)	0.04

Fatty acid (g/100 g)	Capric acid (C10:0)	ND	Stearic acid (C18:0)	0.12	Arachidonic acid (C20:4n6)	0.002
	Undecanoic acid (C11:0)	ND	Oleic acid (C18:1n9)	1.36	Eicosapentaenoic acid (C20:5n3)	ND
	Lauric acid (C12:0)	ND	Elaidic acid (C18:1n9t)	0.002	Heneicosanoic acid (C21:0)	0.004
	Tridecanoic acid (C13:0)	ND	Linoleic acid (C18:2n6c)	4.73	Docosanoic acid (C22:0)	0.004
	Myristic acid (C14:0)	0.005	Translinoleic acid (C18:2n6t)	ND	Erucic acid (C22:1n9)	ND
	Myristoleic acid (C14:1n5)	ND	*α*-Linolenic acid (C18:3n3)	0.098	*cis*-13,16-Docosadienoic acid methyl ester (C22:2)	ND
	Pentadecanoic acid (C15:0)	0.003	*γ*-Linolenic acid (C18:3n6)	0.024	Docosahexaenoic acid (C22:6n3)	0.005
	Pentadecenoic acid (C15:1n5)	ND	Arachidic acid (C20:0)	0.021	Tricosanoic acid (C23:0)	ND
	Hexadecanoic acid (C16:0)	0.74	Paullinic acid (C20:1)	ND	Tetracosanoic acid (C24:0)	0.04
	Palmitoleic acid (C16:1n7)	0.028	Eicosadienoic acid (C20:2)	0.023	Nervonic acid (C24:1n9)	0.041
	Heptadecanoic acid (C17:0)	0.005	Eicosatrienoic acid (C20:3n3)	0.015	Octanoic acid (C8:0)	ND
	Heptadecenoic acid (C17:1n7)	0.003	Dihomo-gamma-linolenic acid (C20:3n6)	ND		

Amino acid (g/kg)	Aspartic acid (Asp)	101.33	Cystine (Cys)	5.25	Phenylalanine (Phe)	8.41
	L-Threonine (Thr)	10.41	Valine (Val)	9.94	Lysine (Lys)	11.01
	Serine (Ser)	9.66	Methionine (Met)	3.62	Histidine (His)	4.83
	Glutamic acid (Glu)	55.98	Isoleucine (Iso)	9.00	Arginine (Arg)	16.39
	Glycine (Gly)	9.22	Leucine (Leu)	14.79	Proline (Pro)	9.17
	Alanine (Ala)	10.58	Tyrosine (Tyr)	6.45		

Minerals	Mercury (Hg)	ND	Zinc (Zn) (mg/kg)	128.3	Copper (Cu) (mg/kg)	33.93
	Lead (Pb) (*μ*g/kg)	2404	Iron (Fe) (mg/kg)	1019	Sodium (Na) (mg/kg)	308.4
	Selenium (Se) (*μ*g/kg)	159.8	Manganese (Mn) (*μ*g/kg)	27010	Potassium (K) (*μ*g/kg)	24240
	Arsenic (As) (*μ*g/kg)	613.3	Chromium (Cr) (*μ*g/kg)	3616		
	Cadmium (Cd) (*μ*g/kg)	2674	Calcium (Ca) (mg/kg)	1206		

ND: nondetected.

**Table 2:** The effects of ME and Sil on the serum and liver levels of AST, ALT, GGT, and ALDH in alcohol-treated mice.

		CTRL	Alcohol	Alcohol + Sil (60 mg/kg)	Alcohol + ME (200 mg/kg)	Alcohol + ME (400 mg/kg)	Alcohol + ME (800 mg/kg)
Serum	AST (U/L)	128.6 ± 1.6	146.2 ± 2.8^#^	134.2 ± 2.7	130.8 ± 7.1	127.5 ± 4.9^∗^	125.0 ± 3.9^∗^
	ALT (U/L)	47.8 ± 0.7	52.8 ± 0.4^#^	48.7 ± 0.7	52.0 ± 1.9	45.4 ± 2.1^∗^	44.0 ± 2.2^∗^
	GGT (ng/L)	67.6 ± 14.9	96.4 ± 8.1^##^	79.2 ± 12.2^∗^	54.0±9.9^∗∗^	71.9 ± 10.6^∗^	63.3 ± 6.7
	ALDH (U/mL)	8.5 ± 0.2	7.7 ± 0.2	8.7 ± 0.1^∗^	8.6 ± 0.4^∗^	8.9 ± 0.3^∗^	8.8 ± 0.4^∗^

Liver	AST (U/g)	56.2 ± 0.6	64.4 ± 1.7^#^	57.3 ± 1.0	61.7 ± 4.9	49.4 ± 2.2^∗^	51.7 ± 2.7^∗^
	ALT (U/g)	17.3 ± 0.3	20.0 ± 0.5^#^	18.2 ± 0.4	21.3 ± 1.4	15.1 ± 1.7^∗^	16.4 ± 1.0^∗^
	GGT (ng/g)	256.9 ± 33.4	397.8 ± 34.3^###^	254.2±43.3^∗∗^	257.2±16.7^∗∗^	303.1 ± 54.3	308.1 ± 17.6
	ALDH (*μ*mol/g)	4.8 ± 0.1	3.5 ± 0.1^##^	5.0±0.1^∗∗^	3.2 ± 0.2	3.8 ± 0.1^∗^	4.0 ± 0.2^∗^

All data are presented as mean ± S.E.M. (*n* = 10). ^#^ *p* < 0.05, ^##^ *p* < 0.01, and ^###^ *p* < 0.001 compared to the control group, ^∗^ *p* < 0.05 and ^∗∗^ *p* < 0.01 compared with the alcohol-treated group.

**Table 3:** The effects of ME and Sil on the serum and liver cytokine levels in alcohol-treated mice.

		CTRL	Alcohol	Alcohol + Sil (60 mg/kg)	Alcohol + ME (200 mg/kg)	Alcohol + ME (400 mg/kg)	Alcohol + ME (800 mg/kg)
Serum	IL-7 (pg/mL)	88.4 ± 2.0	100.7 ± 2.3^#^	95.2 ± 3.0	89.6 ± 2.7^∗^	95.1 ± 3.8	97.4 ± 2.5
	YKL 40 (ng/mL)	52.8 ± 1.3	60.4 ± 2.4^#^	52.9 ± 2.3^∗^	61.9 ± 1.8	50.2 ± 2.0^∗^	55.3 ± 1.6
	PAI-1 (pg/mL)	924.8 ± 14.5	974.5 ± 13.6^#^	915.0 ± 25.7^∗^	730.0 ± 6.0^∗^	843.2 ± 17.0^∗^	860.1 ± 34.0^∗^
	RBP4 (*μ*g/mL)	37.7 ± 0.4	34.4 ± 0.5	36.8 ± 0.8	33.4 ± 1.0	36.3 ± 1.4	37.2 ± 1.7

Liver	IL-7 (pg/mg)	36.7 ± 0.8	41.4 ± 1.3^#^	34.6 ± 1.2^∗^	36.4 ± 1.2^∗^	33.2 ± 1.1^∗^	32.0 ± 1.4^∗^
	YKL 40 (ng/mg)	22.8 ± 0.4	27.1 ± 0.8^#^	23.2 ± 0.7^∗^	21.6 ± 0.8^∗^	20.4 ± 0.6^∗^	21.8 ± 0.8^∗^
	PAI-1 (pg/mg)	295.0 ± 7.3	332.9 ± 9.9^#^	294.8 ± 7.9^∗^	324.0 ± 11.0	291.4 ± 7.0^∗^	299.4 ± 9.6^∗^
	RBP4 (*μ*g/mg)	14.2 ± 0.3	11.3 ± 0.3^##^	15.1±0.3^∗∗^	13.9±0.5^∗∗^	11.2 ± 0.3	12.2 ± 0.3

All data are presented as mean ± S.E.M. (*n* = 10). ^#^ *p* < 0.05 and ^##^ *p* < 0.01 compared with the control group, ^∗^ *p* < 0.05 and ^∗∗^ *p* < 0.01 compared with the alcohol-treated group.

**Table 4:** The effects of ME and Sil on oxidative stress-related factors in the serum and liver tissue of mice.

		CTRL	Alcohol	Alcohol + Sil (60 mg/kg)	Alcohol + ME (200 mg/kg)	Alcohol + ME (400 mg/kg)	Alcohol + ME (800 mg/kg)
Serum	ROS (U/mL)	342.2 ± 5.7	351.3 ± 3.6	330.7 ± 3.4	321.8 ± 10.0	302.5 ± 9.7	301.1 ± 3.8^∗^
	MDA (nmol/mL)	18.5 ± 0.2	20.4 ± 0.5^#^	19.0 ± 0.4	17.7 ± 0.6^∗^	18.0 ± 0.3^∗^	18.2 ± 0.6^∗^
	NO (*μ*mol/L)	28.7 ± 0.8	32.4 ± 0.9^#^	29.6 ± 0.8	35.4 ± 0.7	28.9 ± 0.9^∗^	31.6 ± 0.9
	8-OHdG (ng/L)	40.8 ± 0.9	59.5 ± 1.2^#^	28.8±0.8^∗∗^	38.6 ± 2.1^∗^	55.4 ± 0.5	40.9 ± 1.8^∗^
	SOD (U/mL)	214.7 ± 7.4	167.0 ± 4.7^##^	196.8 ± 5.1^∗^	204.2 ± 6.8^∗^	182.1 ± 3.3^∗^	188.9 ± 5.4^∗^
	GSH-Px (U/mL)	264.6 ± 5.7	239.6 ± 4.7^#^	268.7 ± 5.7^∗^	234.1 ± 7.9	226.3 ± 3.4	268.6 ± 6.5^∗^
	CAT (U/mL)	43.6 ± 0.7	37.9 ± 1.0^#^	44.2 ± 0.5^∗^	53.8 ± 1.2^∗^	42.3 ± 1.5^∗^	42.8 ± 1.0^∗^

Liver	ROS (U/mg)	162.7 ± 2.7	205.4 ± 6.2^##^	184.3 ± 3.2	196.1 ± 12.0	152.7±5.2^∗∗^	151.2±4.0^∗∗^
	MDA (nmol/mg)	5.1 ± 0.1	5.7 ± 0.2^#^	5.5 ± 0.1	6.3 ± 0.3	5.0 ± 0.3^∗^	5.7 ± 0.3
	NO (*μ*mol/g)	14.2 ± 0.2	16.1 ± 0.7^#^	14.5 ± 0.2	16.3 ± 1.5	12.7±0.6^∗∗^	12.1±0.6^∗∗^
	8-OHdG (ng/g)	30.7 ± 2.1	47.9 ± 0.5^#^	27.4 ± 1.1^∗^	30.6 ± 0.6	23.3±1.8^∗∗^	24.7 ± 0.8^∗^
	SOD (U/mg)	91.6 ± 2.0	72.1 ± 2.6^##^	112.5±3.4^∗∗∗^	94.7±5.4^∗∗^	88.6±3.8^∗∗^	109.8±7.4^∗∗∗^
	GSH-Px (U/mg)	101.6 ± 1.3	83.4 ± 1.9^#^	114.8±2.4^∗∗^	124.4±7.8^∗∗^	113.6±4.2^∗∗^	130.7±10.2^∗∗∗^
	CAT (U/mg)	25.0 ± 0.4	20.5 ± 0.5^#^	24.9±0.6^∗∗^	27.7 ± 1.6^∗^	24.6 ± 1.1^∗^	24.1 ± 0.8^∗^

All data are presented as mean ± S.E.M. (*n* = 10). ^#^ *p* < 0.05 and ^##^ *p* < 0.01 compared with the control group, ^∗^ *p* < 0.05, ^∗∗^ *p* < 0.01, and ^∗∗∗^ *p* < 0.001 compared with the alcohol-treated group.

## Data Availability

All generated and analyzed data used to support the findings of this study are included within the article.

## Supplementary Materials

Supplementary Materials For comprehensive analysis, see Tables 1S and 2S and Figure 1S. Table 1S: the effects of ME and Sil on the bodyweight of experimental mice. Table 2S: the effects of ME and Sil on cytokines related to oxidative stress in the spleens of mice with alcohol-induced acute liver injury. Figure 1S: ME alone (800 mg/kg) showed no significant effects on bodyweight or biochemical indexes in serum of healthy mice after 14-day administration. (A) ME alone failed to influence the bodyweight of healthy mice. ME alone showed no effects on the serum levels of (B) AST, (C) ALT, and (D) ALDH in healthy mice. Data expressed as mean ± S.E.M. (*n* = 10) were analyzed using a one-way ANOVA. ME: *M. esculenta*.
