# Supplementary material for: Hepatoprotective Effects of Morchella esculenta against Alcohol-Induced Acute Liver Injury in the C57BL/6 Mouse Related to Nrf-2 and NF-κB Signaling
Source: Oxid Med Cell Longev. 2019 Jul 17;2019:6029876. doi: 10.1155/2019/6029876 (PMC6664553; doi:10.1155/2019/6029876)
Supplement: Supplementary Materials — For comprehensive analysis, see Tables 1S and 2S and Figure 1S. Table 1S: the effects of ME and Sil on the bodyweight of experimental mice. Table 2S: the effects of ME and Sil on cytokines related to oxidative stress in the spleens of mice with alcohol-induced acute liver injury. Figure 1S: ME alone (800 mg/kg) showed no significant effects on bodyweight or biochemical indexes in serum of healthy mice after 14-day administration. (A) ME alone failed to influence the bodyweight of healthy mice. ME alone showed no effects on the serum levels of (B) AST, (C) ALT, and (D) ALDH in healthy mice. Data expressed as mean ± S.E.M. (n = 10) were analyzed using a one-way ANOVA. ME: M. esculenta. [file 6029876.f1.pdf]

Supplementary Materials

Results

ME showed significant effects on the bodyweights of acute alcohol-exposed mice

During the two-week trial period, the weight of mice in the alcohol group was decreased upto 8.3% compared with that of the control group. ME treatment significantly enhanced the bodyweights of acute alcohol-exposed mice but not Sil. (Table. 1S). ME alone showed no significant effects on the bodyweight of heathy mice, indicating the safe using of ME (Figure.1S A).

ME showed significant effects on cytokines related to oxidative stress in spleen of alcohol-treated mice

Compared with acute alcohol-exposed mice, two weeks of ME treatment strongly reduced the levels of ROS, MDA and NO, and enhanced the levels of SOD, GSH-Px and CAT in spleen of mice with acute alcohol injury (P< 0.05; Table. 2S).

The effect of ME on AST, ALT and ALDH

Compared with normal control group, ME alone showed no significant effects on the levels of AST, ALT and ALDH in serum of healthy mice (Fig. 2S B-D).

Table. 1S The effects of ME and Sil on bodyweight of experimental mice

	CTRL	Alcohol	Alcohol + Sil (60 mg/kg)	Alcohol + ME (200 mg/kg)	Alcohol + ME (400 mg/kg)	Alcohol + ME (800 mg/kg)
1st day	21.9 ± 0.2	22.4 ± 0.2	22.5 ± 0.3	21.6 ± 0.5	22.6 ± 0.4	22.7±0.3
3th day	21.7 ± 0.2	19.9 ± 0.2	19.4 ± 0.2	19.1 ± 0.3	19.8 ± 0.5	20.7±0.3
6th day	21.5 ± 0.3	17.6 ± 0.3#	18.6 ± 0.3	16.8 ± 0.3	20.6 ± 0.4*	20.5±0.5*
9st day	20.2 ± 0.3	18.2 ± 0.3#	18.9 ± 0.4	18.2 ± 0.6	20.0 ± 0.5*	20.3±0.4*
12st day	21.6 ± 0.3	19.8 ± 0.4#	20.3 ± 0.4	19.7 ± 0.6	21.4 ± 0.7*	20.1±0.4

Date were presented as mean ± S.E.M. (n=10). # P<0.05 compared with control group, * P<0.05 compared with alcohol only treated mice. ME: *M. esculenta*; Sil: silybin.

Table. 2S The effects of ME and Sil on cytokines related to oxidative stress in the spleens of mice with alcohol-induced acute liver injury.

	CTRL	Alcohol	Alcohol + Sil (60 mg/kg)	Alcohol + ME (200 mg/kg)	Alcohol + ME (400 mg/kg)	Alcohol + ME (800 mg/kg)
ROS (U/mg)	209.9 ± 3.3	305.4 ± 9.2##	243.6 ± 11.0**	241.5 ± 19.8*	198.4 ±12.6**	158.4 ± 5.0***
MDA (nmol/mg)	8.0 ± 0.1	10.9 ± 0.4##	9.2 ± 0.3*	8.8 ± 0.7*	8.0 ± 0.6**	8.0 ± 0.5**
NO (µmol/g)	14.1 ± 0.3	16.7 ± 0.8#	17.0 ± 0.3	15.4 ± 0.6	14.2 ± 0.7*	14.0 ± 0.8*
SOD (U/mg)	161.4 ± 3.8	124.2 ± 4.5##	156.2 ± 3.0**	155.3 ± 8.8**	140.9 ± 6.9*	153.5 ± 7.8**
GSH-Px (U/mg)	260.3 ± 5.3	226.2 ± 12.5#	266.2 ± 9.3*	234.3 ± 10.9	230.3 ± 15.5	251.7 ± 12.1*
CAT(U/mg)	23.5 ± 0.6	22.5 ± 2.0	25.5 ± 0.8	30.1 ± 3.0**	20.0 ± 1.5	21.6 ± 1.5

Date were presented as mean ± S.E.M. (n=10). #P< 0.05 and ## P< 0.01 compared with control group, * P< 0.05, **P< 0.01 and *** P<0.001 compared with alcohol only treated mice. ME: *M. esculenta*; Sil: silybin.

Figure List

Figure 1S. ME alone (800 mg/kg) showed no significant effects on bodyweight or biochemical indexes in serum of healthy mice after 14-day administration. (**A**) ME alone failed to influence the bodyweight of healthy mice. ME alone showed no effects on the serum levels of (**B**) AST, (**C**) ALT and (**D**) ALDH in healthy mice. Data expressed as mean ± S.E.M. (n=10) were analyzed using a one-way ANOVA. ME: *M. esculenta*.

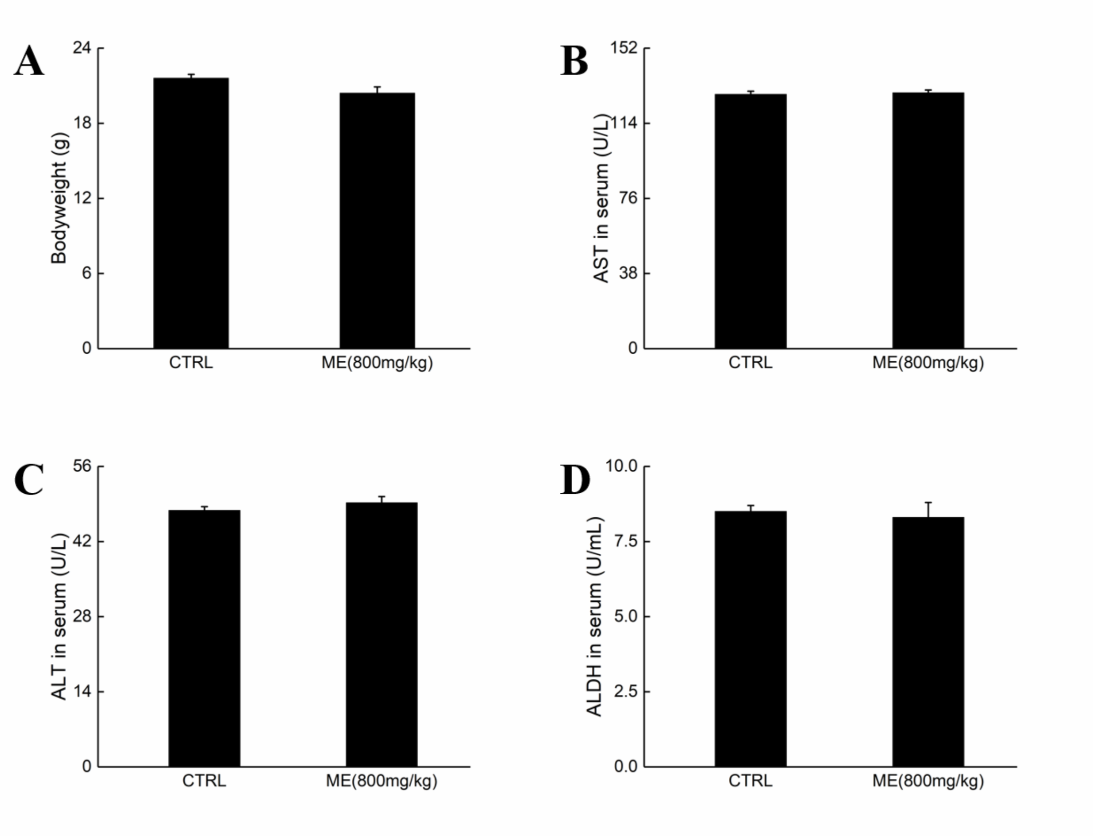

Figure.1S
